# Enhancing Ophthalmic Diagnosis and Treatment with Artificial Intelligence

**DOI:** 10.3390/medicina61030433

**Published:** 2025-02-28

**Authors:** David B. Olawade, Kusal Weerasinghe, Mathugamage Don Dasun Eranga Mathugamage, Aderonke Odetayo, Nicholas Aderinto, Jennifer Teke, Stergios Boussios

**Affiliations:** 1 Department of Allied and Public Health, School of Health, Sport and Bioscience, University of East London, London E16 2RD, UK; 2 Department of Research and Innovation, Medway NHS Foundation Trust, Gillingham ME7 5NY, UK; kusal.weerasinghe@nhs.net (K.W.); j_teke@nhs.net (J.T.); stergiosboussios@gmail.com (S.B.); 3 Department of Public Health, York St John University, London YO31 7EX, UK; 4 School of Health and Care Management, Arden University, Arden House, Middlemarch Park, Coventry CV3 4FJ, UK; 5 National Eye Hospital, Colombo 01000, Sri Lanka; dasun1000@gmail.com; 6 School of Nursing, Tung Wah College, Hong Kong SAR, China; ronkeodetayo@gmail.com; 7 Department of Medicine and Surgery, Ladoke Akintola University of Technology, Ogbomoso 210214, Nigeria; nicholasoluwaseyi6@gmail.com; 8 Faculty of Medicine, Health and Social Care, Canterbury Christ Church University, Canterbury CT1 1QU, UK; 9 School of Cancer & Pharmaceutical Sciences, King’s College London, Strand, London WC2R 2LS, UK; 10 Kent Medway Medical School, University of Kent, Canterbury CT2 7NZ, UK; 11 Department of Medical Oncology, Medway NHS Foundation Trust, Gillingham ME7 5NK, UK; 12 AELIA Organization, 57001 Thessaloniki, Greece

**Keywords:** artificial intelligence, ophthalmology, machine learning, diabetic retinopathy, age-related macular degeneration, glaucoma

## Abstract

The integration of artificial intelligence (AI) in ophthalmology is transforming the field, offering new opportunities to enhance diagnostic accuracy, personalize treatment plans, and improve service delivery. This review provides a comprehensive overview of the current applications and future potential of AI in ophthalmology. AI algorithms, particularly those utilizing machine learning (ML) and deep learning (DL), have demonstrated remarkable success in diagnosing conditions such as diabetic retinopathy (DR), age-related macular degeneration, and glaucoma with precision comparable to, or exceeding, human experts. Furthermore, AI is being utilized to develop personalized treatment plans by analyzing large datasets to predict individual responses to therapies, thus optimizing patient outcomes and reducing healthcare costs. In surgical applications, AI-driven tools are enhancing the precision of procedures like cataract surgery, contributing to better recovery times and reduced complications. Additionally, AI-powered teleophthalmology services are expanding access to eye care in underserved and remote areas, addressing global disparities in healthcare availability. Despite these advancements, challenges remain, particularly concerning data privacy, security, and algorithmic bias. Ensuring robust data governance and ethical practices is crucial for the continued success of AI integration in ophthalmology. In conclusion, future research should focus on developing sophisticated AI models capable of handling multimodal data, including genetic information and patient histories, to provide deeper insights into disease mechanisms and treatment responses. Also, collaborative efforts among governments, non-governmental organizations (NGOs), and technology companies are essential to deploy AI solutions effectively, especially in low-resource settings.

## 1. Introduction

Ophthalmology, the branch of medicine dedicated to studying and treating disorders and diseases of the eye and visual system, stands at the forefront of medical innovation. Over the past few decades, technological advancements have significantly transformed the field, enhancing diagnostic accuracy, therapeutic outcomes, and overall patient care [1,2,3]. One of the most impactful technological advancements in recent years is artificial intelligence (AI), which encompasses both machine learning (ML) and deep learning (DL). AI involves the simulation of human intelligence processes by machines, particularly computer systems. These processes include learning (the acquisition of information and rules for using the information), reasoning (using rules to reach approximate or definite conclusions), and self-correction [4,5]. ML, a subset of AI, enables systems to learn and improve from experience without being explicitly programmed [6]. DL, a further subset of ML, utilizes neural networks with multiple layers to analyze various factors of data [7]. In ophthalmology, AI technologies have shown significant promise in transforming traditional practices. This transformation is driven by the field’s heavy reliance on imaging and diagnostic data, which are well suited for AI applications [4,8]. The ability of AI to process and analyze vast amounts of data rapidly and accurately positions it as a revolutionary tool in eye care. For example, a deep learning algorithm has been successfully used to analyze retinal images from a fundus camera to detect early signs of diabetic retinopathy in clinical settings, while machine learning models applied to OCT images have identified subtle retinal changes in patients with AMD [9,10]. These AI applications are highly dependent on the quality and consistency of imaging devices, such as fundus cameras and OCT devices, though they are increasingly integrated with surgical systems like phaco machines to enhance procedural safety [9,11].

One of the most profound impacts of AI in ophthalmology is in the realm of diagnostics. AI systems, particularly those leveraging DL techniques such as convolutional neural networks (CNNs), are adept at recognizing complex patterns in imaging data, which is crucial for diagnosing various eye conditions [7,12]. Diabetic retinopathy (DR) is a significant cause of blindness among working-age adults worldwide [13]. Traditional diagnostic methods rely on the manual examination of retinal images, which can be time-consuming and subject to human error. AI algorithms, however, have demonstrated the ability to detect DR with high sensitivity and specificity [9,10]. Notably, the study by Gulshan et al. highlighted an AI system capable of identifying DR in retinal images with performance comparable to that of experienced ophthalmologists [10]. Such advancements ensure that patients at risk of vision loss are identified and treated promptly, thus preventing disease progression. Age-related macular degeneration (AMD) is another major cause of vision loss, particularly among the elderly. Early detection and monitoring are critical in managing AMD, and AI has proven to be a valuable tool in this regard [11]. AI models have been developed to analyze optical coherence tomography (OCT) images, differentiating between normal and pathological features with remarkable accuracy. For instance, research has shown that AI algorithms can classify AMD stages from OCT scans, providing crucial support for early intervention and personalized treatment strategies [11,14].

Glaucoma, often referred to as the “silent thief of sight”, is characterized by optic nerve damage and is a leading cause of irreversible blindness [15,16]. Early detection and continuous monitoring are essential for managing glaucoma. AI applications, particularly those involving the automated analysis of OCT images and visual field tests, have shown significant promise [17,18]. In the context of glaucoma, while traditional trend analysis of visual field parameters relies on observing longitudinal changes, AI-based prediction leverages complex patterns from multimodal data to forecast progression with greater sensitivity and specificity [17]. These AI tools assist ophthalmologists in detecting early signs of glaucoma and tracking disease progression, facilitating timely and effective interventions.

In addition to its diagnostic capabilities, AI is revolutionizing the treatment and management of ophthalmic diseases. Personalized medicine, which tailors treatment plans to individual patient profiles, is significantly enhanced by AI’s ability to analyze extensive datasets and predict treatment outcomes [19,20]. AI can analyze data from diverse sources, including patient demographics, medical history, genetic information, and imaging data, to develop personalized treatment plans [19]. In the context of ophthalmology, this capability is particularly valuable. For example, AI-driven models can predict which patients with DR are likely to respond to specific treatments, enabling more targeted and effective interventions [9]. Moreover, advancements in AI-guided surgical tools—such as those that stabilize the anterior chamber during cataract surgery or automate instrument calibration in vitreoretinal procedures—have already been incorporated into routine practice, subtly enhancing the ease and safety of surgical procedures [21].

AI is not only transforming clinical practice but also enhancing service delivery in ophthalmology. By automating routine tasks and optimizing workflows, AI can significantly improve efficiency and patient care [21,22]. AI-powered screening programs are being implemented to improve access to eye care [23,24], especially in remote and underserved areas [25]. Mobile applications and teleophthalmology services utilize AI to screen for common eye diseases, such as DR and glaucoma [26,27], facilitating early detection and timely referral to specialists. These programs are particularly beneficial in addressing disparities in eye care access, ensuring that more people receive the necessary care. In clinical settings, AI can streamline workflows by automating routine tasks, such as image analysis and patient triage [9,22]. This not only enhances efficiency but also allows ophthalmologists to focus on more complex cases, thereby improving the overall quality of care. AI-driven systems can prioritize patients based on the severity of their conditions, ensuring that those in urgent need receive prompt attention [20].

The rationale for this study is grounded in the transformative potential of AI to revolutionize ophthalmic practices. Traditional diagnostic methods in ophthalmology, often reliant on subjective interpretation and manual analysis, face limitations in terms of efficiency and accuracy [9]. With the rising prevalence of eye diseases like DR, AMD, and glaucoma, there is an urgent need for advanced diagnostic and treatment tools. AI-powered screening programs and teleophthalmology services present significant opportunities to address the global burden of eye diseases [26].

The objectives of this study are multifaceted, aiming to provide a comprehensive overview of AI applications in ophthalmology, assess the benefits and challenges of integrating AI into clinical practice, and explore future directions for AI-driven advancements in the field. The main objective of this narrative review is to systematically evaluate the current landscape of AI technologies in ophthalmology by addressing the following research questions: (1) What are the specific AI tools and techniques currently employed in ophthalmic diagnosis, treatment, and service delivery? (2) What benefits and challenges are associated with their integration into clinical practice? (3) What future trends and directions can be anticipated for AI-driven innovations in this field?

**Figure 1:**
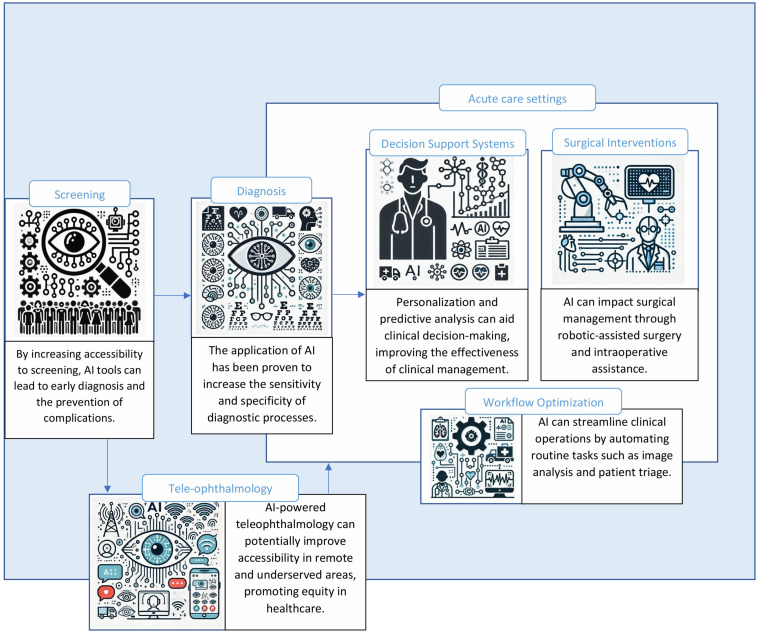
AI applications in various ophthalmology domains.

The novelty of this review lies in its holistic approach, examining not only individual AI tools and techniques but also their integration across various data modalities to offer more comprehensive insights into eye diseases and treatments. Furthermore, the study emphasizes the role of AI in personalizing treatment plans, improving service delivery through teleophthalmology, and addressing ethical and practical challenges associated with AI implementation. By highlighting emerging trends and potential advancements, this study aims to provide a forward-looking perspective that can inform both clinical practice and future research, ultimately contributing to the responsible and effective use of AI in ophthalmology. Figure 1 below shows various applications of AI in ophthalmology domains.

## 2. Methodology

This narrative review employs a comprehensive literature review methodology to gather, analyze, and synthesize existing research on the applications of AI in ophthalmology. The methodology includes several key steps: defining the research scope, selecting relevant databases, establishing inclusion and exclusion criteria, conducting a systematic search, and analyzing and synthesizing the collected data.

### 2.1. Database Selection and Search Strategy

Relevant peer-reviewed articles, conference papers, and review articles were identified using multiple scientific databases, including PubMed, Google Scholar, IEEE Xplore, and ScienceDirect. The search strategy involved the use of keywords and phrases such as “AI in ophthalmology”, “machine learning in eye care”, “deep learning in ophthalmic diagnosis”, “AI in diabetic retinopathy”, “AI in glaucoma detection”, “teleophthalmology”, and “AI in personalized medicine for eye diseases”. Boolean operators (AND, OR) were employed to refine and expand the search to capture a comprehensive set of relevant studies.

### 2.2. Inclusion and Exclusion Criteria

To ensure the relevance and quality of the reviewed literature, specific inclusion and exclusion criteria were established. The included studies were those that met the following criteria:

- Focus on the application of AI in ophthalmology.
- Are peer-reviewed and published within the last ten years (2013–2023) to ensure contemporary relevance.
- Provide empirical data, case studies, systematic reviews, or meta-analyses on the use of AI in diagnosing, treating, or managing eye diseases.
- Discuss ethical, practical, or future-oriented aspects of AI in ophthalmology.

Studies were excluded if they met the following criteria:

- Do not specifically address AI applications in ophthalmology.
- Are opinion pieces, editorials, or anecdotal reports without empirical data.
- Are published in non-English languages, due to language constraints.

### 2.3. Systematic Search and Data Extraction

A systematic search of the selected databases was conducted using predefined keywords and criteria. Titles and abstracts of the retrieved articles were screened for relevance, and full-text versions of potentially relevant articles were obtained for detailed review. A data extraction form was used to systematically collect information from each included study, including study objectives, methods, AI techniques used, findings, benefits, challenges, and future recommendations.

### 2.4. Data Analysis and Synthesis

The extracted data were analyzed using qualitative synthesis methods. The studies were categorized based on their primary focus: diagnostic applications, therapeutic applications, service delivery improvements, and ethical considerations. Within each category, thematic analysis was conducted to identify common themes, trends, and gaps in the literature. The findings were then synthesized to provide a comprehensive overview of the current state of AI in ophthalmology, highlighting key advancements, benefits, challenges, and future directions.

## 3. AI in Ophthalmic Diagnosis

Table 1 below highlights the diverse applications of AI in ophthalmic diagnosis, emphasizing the technology, key systems, accuracy, clinical applications, advantages, and challenges for various eye conditions. AI algorithms for glaucoma predict disease progression and assist in timely intervention [28]. ML models streamline cataract diagnosis by automating assessment and grading from slit-lamp images [29,30]. DL models for retinal vein occlusion (RVO) enhance diagnostic precision, reducing manual analysis [31,32]. AI integration of genetic data with imaging for retinitis pigmentosa offers comprehensive disease insights [33,34]. AI-powered corneal topography for keratoconus ensures early detection and better treatment outcomes [35]. AI applications in ocular surface diseases improve patient management and reduce workload [36]. For uveitis, AI combines imaging and clinical data for early detection and tailored treatment plans [37,38]. Despite these advancements, challenges like data privacy, algorithmic bias, workflow integration, and model generalizability persist across applications [39].

### 3.1. Diabetic Retinopathy

DR is a leading cause of blindness worldwide, primarily affecting individuals with diabetes [40,41]. It is characterized by damage to the blood vessels of the retina, which can lead to vision impairment and, ultimately, blindness if left untreated. Early detection and timely intervention are crucial to preventing vision loss [40]. However, traditional methods for diagnosing DR rely on manual examination of retinal images by ophthalmologists, which can be time-consuming and prone to variability [33,40].

AI algorithms, especially CNNs, have demonstrated remarkable accuracy in detecting DR from retinal images. CNNs are well suited for image analysis tasks due to their ability to automatically learn and extract features from raw image data, enabling them to identify patterns and anomalies indicative of DR [42,43]. Numerous studies have shown that AI can achieve sensitivity and specificity comparable to, or even surpass, human experts in detecting DR [10,42,43,44,45]. One of the most notable AI systems for DR detection is the EyeArt AI system. EyeArt has been extensively validated in clinical settings and has shown over 90% sensitivity in detecting referable DR, which refers to cases that require further evaluation by an ophthalmologist [42,46]. A pivotal study involving EyeArt demonstrated its ability to accurately identify DR in a diverse patient population, highlighting its potential as a reliable screening tool [46]. However, it is important to note that according to WHO criteria for a valid screening program, a system must achieve at least ≥80% sensitivity and ≥95% specificity; while EyeArt demonstrates sensitivity over 90%, its specificity has been reported in some studies to fall below the 95% threshold [42,46]. The system’s high sensitivity ensures that most cases of referable DR are detected, thereby reducing the risk of missed diagnoses and facilitating early intervention.

Another prominent AI model is the system developed by Google Health, which utilizes DL techniques to analyze retinal photographs [10,12]. This AI model has been trained on a large dataset of retinal images labeled by expert ophthalmologists. In a recent study, the Google Health model achieved sensitivity and specificity rates comparable to those of board-certified ophthalmologists [10,47]. The model’s ability to accurately identify both referable and non-referable DR underscores its potential to enhance screening programs and improve access to eye care, particularly in resource-limited settings.

Comparative studies have further underscored the efficacy of AI in DR detection. For example, a study comparing the performance of several AI models, including EyeArt and Google Health’s system, found that these AI tools consistently outperformed traditional manual grading by ophthalmologists in terms of both speed and accuracy [12,42,45]. These findings suggest that AI can serve as a valuable adjunct to human expertise, enabling more efficient and reliable screening processes. In real-world applications, AI systems for DR detection have been deployed in various settings, from urban hospitals to rural clinics [12,46]. The scalability and accessibility of AI technologies make them particularly advantageous for large-scale screening programs. For instance, in India, where the prevalence of diabetes is high and access to specialized eye care is limited, AI-based screening initiatives have been implemented to identify patients at risk of DR [47]. These programs leverage AI to analyze retinal images captured by mobile screening units, providing immediate feedback and referral recommendations [10,43]. The integration of AI in such programs has demonstrated significant improvements in screening coverage and diagnostic accuracy, ultimately contributing to better patient outcomes.

### 3.2. Age-Related Macular Degeneration

AMD is a leading cause of vision loss, particularly among older adults. It affects the macula, the part of the retina responsible for central vision, leading to progressive vision impairment and, in severe cases, blindness [48]. Early and accurate diagnosis is crucial for managing AMD and preventing severe visual deterioration. Traditional diagnostic methods include clinical examination and imaging techniques, such as OCT [49,50]. However, these methods can be time-consuming and require significant expertise.

AI systems have been developed to classify AMD stages from OCT images with remarkable accuracy. These systems utilize DL algorithms, such as CNNs, to analyze the intricate details of retinal images and distinguish between normal and pathological features [45,51]. By processing large volumes of data, AI models can learn to identify the subtle signs of early AMD, intermediate stages, and advanced forms of the disease, including both dry and wet AMD [11,52]. In clinical validation studies, the DL system achieved diagnostic accuracy comparable to that of experienced retinal specialists [12,53]. This level of precision highlights the potential of AI to assist in early detection and monitoring, enabling timely interventions that can slow disease progression and preserve vision.

The integration of AI into AMD diagnosis provides substantial support to ophthalmologists. AI models can rapidly analyze OCT scans and flag images that exhibit signs of AMD, prioritizing patients who need immediate attention [44,50]. This triage capability reduces the diagnostic burden on ophthalmologists, allowing them to focus on patients with more complex cases [11,52]. For instance, studies have shown that AI-driven analysis of OCT images can significantly decrease the time required for initial screenings, freeing up resources and improving the efficiency of eye care services [12,54]. Additionally, AI systems can provide continuous monitoring for patients with AMD. By comparing sequential OCT images, AI can detect subtle changes that may indicate disease progression or response to treatment [52,55].

In real-world clinical settings, AI systems for AMD detection and classification have shown promising results. For example, the Moorfields–DeepMind collaboration developed an AI system capable of diagnosing a wide range of retinal diseases, including AMD, from OCT scans [12,56]. The system was tested in clinical practice and demonstrated high accuracy, often exceeding that of human experts. This collaboration highlighted the potential for AI to be integrated into routine clinical workflows, providing reliable and scalable diagnostic support. Another significant application is the use of AI in large-scale screening programs. In regions with limited access to specialized eye care, AI-powered screening tools can facilitate early detection of AMD and other retinal diseases [11,57]. Mobile screening units equipped with OCT devices and AI analysis capabilities can reach underserved populations, providing immediate feedback and referral recommendations [58,59]. These programs have shown success in identifying individuals at risk and ensuring they receive timely and appropriate care.

### 3.3. Glaucoma

Glaucoma is a group of eye conditions characterized by damage to the optic nerve, often associated with elevated intraocular pressure [16,60]. It is one of the leading causes of irreversible blindness worldwide [60,61]. Early detection and continuous monitoring are crucial to prevent significant vision loss, as the damage caused by glaucoma is typically asymptomatic in the early stages. AI applications have shown great promise in improving the diagnosis and management of glaucoma through the automated analysis of OCT images and visual field tests [62,63]. Studies have demonstrated that AI systems can reliably identify glaucomatous changes in the optic nerve head and retinal nerve fiber layer [28,64]. For instance, an AI model developed by researchers at Moorfields Eye Hospital and DeepMind was trained on a large dataset of OCT images and demonstrated performance comparable to that of expert ophthalmologists in diagnosing glaucoma [12,55]. This model could detect structural abnormalities associated with glaucoma and provide diagnostic suggestions with high sensitivity and specificity. By automating the detection of glaucoma-related changes, AI systems can assist ophthalmologists in identifying patients at risk of glaucoma earlier than conventional methods [28,63].

Visual field testing is another essential component in diagnosing and monitoring glaucoma. It measures a patient’s peripheral vision and helps identify functional loss caused by optic nerve damage [65,66]. Traditional visual field tests often rely on trend analysis—using statistical methods such as linear regression to evaluate changes in visual field parameters over time—which can be subjective and influenced by patient performance, leading to variability in results. In contrast, AI-based prediction leverages complex pattern recognition from the entire dataset, including nonlinear trends, to forecast disease progression with greater sensitivity and specificity [63,67]. ML algorithms can analyze patterns in visual field data to detect early glaucomatous changes and predict disease progression [68]. One significant study by Medeiros et al. demonstrated that an AI algorithm could predict the future progression of visual field loss in glaucoma patients with high accuracy [69]. The algorithm analyzed longitudinal visual field data, identifying patterns that indicated the likelihood of disease progression. This predictive capability allows for more proactive management of glaucoma, enabling timely interventions to prevent further vision loss.

AI tools for glaucoma diagnosis and monitoring have been validated in clinical settings, showing substantial benefits in improving patient care [12,17]. For example, AI-powered platforms that integrate OCT analysis and visual field data have been implemented in ophthalmology clinics to assist clinicians in making more informed decisions [69]. These platforms provide a comprehensive assessment of glaucoma, combining structural and functional data to offer a holistic view of the disease [18,61]. In community screening programs, AI systems have been used to identify individuals at risk of glaucoma, particularly in underserved areas where access to specialist eye care is limited [25,70]. This approach has been shown to increase the detection rates of glaucoma and facilitate earlier treatment.

## 4. AI in Treatment and Management

### 4.1. Personalized Treatment Plans

AI models, particularly those based on ML and DL techniques, have shown remarkable ability in predicting disease progression and treatment outcomes. By analyzing diverse data sources such as patient demographics, genetic information, imaging data, and treatment histories, AI can generate precise predictions tailored to individual patients [22,71]. Researchers have developed models that analyze retinal images and other relevant data to forecast the likelihood of disease worsening [9,10,45]. These predictions enable ophthalmologists to tailor treatment plans according to the specific needs of each patient, such as adjusting the frequency of monitoring visits or the type of interventions.

Similarly, in AMD management, AI-driven models can analyze OCT images along with clinical data to predict how patients will respond to various treatments, such as anti-vascular endothelial growth factor (anti-VEGF) injections [11,51,72]. Studies have shown that AI can identify patients who are likely to benefit from specific therapies and those who may require alternative treatment strategies [1,22,57]. This capability allows for more targeted and effective interventions, improving patient outcomes and reducing unnecessary treatments.

AI’s ability to analyze complex datasets and recognize patterns that may not be apparent to human clinicians plays a crucial role in optimizing therapeutic strategies. By continuously learning from new data, AI models can refine their predictions and recommendations, ensuring that treatment plans remain up to date with the latest clinical insights and patient responses [12,22,71]. AI models can predict which patients are at higher risk of rapid disease progression and require more aggressive treatment, such as early surgical intervention, versus those who can be managed with less intensive therapies [58,73]. This individualized approach helps in allocating resources more efficiently and improving the quality of care.

The application of AI in personalizing treatment plans has shown promising results in improving patient outcomes and reducing treatment costs. By accurately predicting disease progression and tailoring interventions, AI helps in achieving better clinical outcomes with fewer complications [45]. Early and precise interventions can prevent the progression of eye diseases, reducing the need for more extensive and costly treatments later on. In addition to clinical benefits, AI-driven personalized treatment plans can lead to significant cost savings for healthcare systems [45,74]. By optimizing the use of resources and minimizing unnecessary treatments, AI contributes to more efficient healthcare delivery. For instance, AI models can help determine the optimal frequency of anti-VEGF injections for AMD patients, reducing the number of injections needed while maintaining therapeutic efficacy [52,72]. This not only lowers treatment costs but also improves patient compliance and satisfaction.

### 4.2. Surgical Applications

AI is transforming ophthalmic surgery by enhancing precision, reducing complications, and improving patient outcomes through robotic-assisted surgery and AI-guided instruments [75,76,77]. These advanced technologies provide real-time feedback, assist in making accurate incisions, and optimize various surgical procedures, such as cataract surgery [77,78]. Traditional cataract surgery involves manually removing the cloudy lens and replacing it with an artificial lens. This procedure requires steady hands and precise movements, as even minor deviations can lead to complications [78]. Robotic-assisted surgery is revolutionizing the field of ophthalmology by providing surgeons with enhanced control and precision. AI-powered robotic systems can assist in performing intricate surgical tasks that require a high degree of accuracy [79,80]. One of the key advantages of robotic-assisted surgery is its ability to reduce human error [76,81]. Robotic systems equipped with AI algorithms can stabilize the surgical instruments and perform precise maneuvers, minimizing the risk of errors. In addition, modern phaco devices are designed to automatically regulate fluidics to maintain a stable anterior chamber during cataract surgery, which reduces intraoperative fluctuations and protects the corneal endothelium. Furthermore, features such as real-time intraoperative imaging overlays and automated fluid management systems have been seamlessly integrated into everyday surgical practice, providing subtle yet significant improvements that enhance surgical safety and ease without drawing undue attention from the surgeon.

AI-guided instruments are another significant innovation in ophthalmic surgery. For example, in retinal surgery, AI-guided instruments can assist surgeons in making precise incisions and accurately positioning implants [75,82]. AI algorithms can analyze intraoperative data, such as imaging and sensor information, to provide surgeons with critical insights [77,80]. This real-time analysis helps in identifying optimal incision sites, avoiding critical structures, and ensuring proper alignment of surgical instruments.

Cataract surgery is one of the most common ophthalmic procedures, and AI is playing a crucial role in enhancing its precision and outcomes. AI-powered robotic systems can assist in various stages of cataract surgery, from preoperative planning to intraoperative execution [77,82]. Preoperative planning involves creating a detailed map of the patient’s eye to guide the surgical procedure [75]. AI algorithms can analyze diagnostic images and generate a precise surgical plan, including the optimal size and location of the incisions and the appropriate power and position of the intraocular lens [83,84]. This personalized approach ensures that the surgery is tailored to the individual patient’s anatomy, leading to better visual outcomes. During the surgery, AI-guided instruments provide real-time feedback to the surgeon, helping them make accurate incisions and perform delicate maneuvers [85]. For instance, femtosecond laser-assisted cataract surgery utilizes AI to control the laser, making precise corneal incisions and fragmenting the cataract with high accuracy [82,85]. This technology reduces the risk of complications, such as capsular tears and corneal astigmatism, and shortens the recovery time for patients.

AI-assisted ophthalmic surgery offers several benefits in terms of reducing complications and improving recovery times. By enhancing surgical precision and minimizing human error, AI technologies help lower the incidence of postoperative complications [81,84]. Moreover, AI can contribute to faster recovery times by ensuring that surgical procedures are performed with minimal trauma to the surrounding tissues [75]. Precise incisions and optimized surgical techniques reduce inflammation and promote quicker healing [85]. Patients undergoing AI-assisted cataract surgery often experience faster visual recovery and improved overall satisfaction.

## 5. AI in Ophthalmology Service Delivery

AI is revolutionizing service delivery in ophthalmology by automating routine tasks, optimizing workflows, and enhancing efficiency in patient care [86]. These advancements are particularly impactful in screening programs, where AI-powered solutions improve access to eye care, especially in remote and underserved areas [8,87]. Table 2 below outlines various AI applications in ophthalmology service delivery, detailing the technologies, key systems, clinical applications, advantages, and challenges associated with each area, including screening programs, teleophthalmology, workflow optimization, patient monitoring, decision support systems, resource allocation, and patient engagement.

### 5.1. Screening Programs

AI-powered screening programs are transforming the landscape of ophthalmic care by enabling large-scale, efficient, and accurate screening processes. These programs utilize advanced algorithms to analyze retinal images and other relevant data, identifying signs of eye diseases with high accuracy [8,87]. The implementation of AI in screening programs offers several benefits, including improved access to care, early detection, and reduced healthcare costs [88,89]. Mobile applications and teleophthalmology services are at the forefront of AI-driven screening programs. These platforms allow for remote screening of eye diseases, making it possible to reach populations that lack access to traditional eye care services [26,90]. AI algorithms integrated into mobile apps can analyze images captured by smartphone cameras or portable retinal imaging devices, providing immediate diagnostic feedback.

Teleophthalmology services extend the reach of AI-powered screening by connecting patients in remote areas with specialists in urban centers [26,91]. Retinal images and other relevant data are transmitted to centralized AI systems for analysis. The results are then reviewed by ophthalmologists who can provide detailed assessments and recommendations [8,90]. This model has been successfully implemented in various regions, significantly reducing the barriers to accessing specialized eye care.

In many parts of the world, there is a shortage of trained ophthalmologists, and patients in rural or underserved areas often face long travel distances to receive care. AI-driven screening tools can bridge this gap by providing accurate and timely diagnoses at the point of care [84,87]. One notable example is the implementation of AI screening programs in India, where DR is a major public health concern [45,47,92]. AI-powered systems have been integrated into primary care settings, allowing healthcare workers to screen patients and refer those with positive findings to specialized eye care centers [22,84,91]. This approach has significantly increased the detection rates of DR and reduced the burden on tertiary care centers.

The automation of routine screening tasks through AI not only improves efficiency but also reduces healthcare costs by reducing the need for unnecessary referrals and follow-up visits [21,71,89]. By accurately identifying patients who need further evaluation, AI systems help ensure that healthcare resources are used more effectively. This targeted approach minimizes the financial burden on both healthcare systems and patients. Furthermore, AI-powered screening programs can integrate with electronic health records (EHR) systems, streamlining the documentation and management of patient data [22,71]. Automated data entry and analysis reduce administrative workloads, allowing healthcare providers to allocate more time to patient care.

The real-world application of AI in ophthalmology service delivery has yielded numerous success stories. For example, the EyePACS program in the United States uses AI to screen for DR in underserved populations [26,93]. This program has successfully screened millions of patients, identifying those at risk and facilitating timely treatment. The AI system used in EyePACS has demonstrated high accuracy, comparable to that of human graders, highlighting the potential of AI to enhance screening programs on a large scale [42]. Another success story comes from the United Kingdom, where the National Health Service (NHS) has implemented AI-powered screening for DR [8,23]. The AI system analyzes retinal images and flags those that require further review by an ophthalmologist. This approach has improved the efficiency of the screening process and ensured that patients with sight-threatening retinopathy receive prompt care.

### 5.2. Workflow Optimization

AI is significantly enhancing workflow optimization in ophthalmology by automating routine tasks, such as image analysis and patient triage [9,84,94]. This not only improves efficiency but also allows ophthalmologists to focus on more complex cases, ultimately enhancing overall service delivery. One of the key areas where AI is making a substantial impact is in the automation of routine tasks. Image analysis, a critical component of ophthalmic diagnostics, can be time-consuming and labor-intensive. AI algorithms, particularly those based on deep learning, can analyze large volumes of imaging data quickly and accurately [4,73].

By automating image analysis, AI significantly reduces the workload of ophthalmologists and technicians [1,84]. This not only speeds up the diagnostic process but also ensures a high level of consistency and accuracy in the interpretation of imaging data. Studies have shown that AI algorithms can match or even exceed the diagnostic accuracy of human experts in detecting various eye conditions [3,12,84,90]. This allows for a more efficient allocation of human resources, enabling clinicians to dedicate their time and expertise to more complex and critical cases.

Patient triage is another area where AI is optimizing clinical workflows. Efficient triage systems are essential for prioritizing patients based on the urgency and severity of their conditions. AI-driven triage systems use advanced algorithms to analyze patient data, including medical histories, symptoms, and imaging results, to determine the urgency of each case [22,71]. This prioritization ensures that patients with severe conditions receive timely care, reducing the risk of complications and improving overall outcomes. AI-based triage systems can also provide decision support to clinicians, offering recommendations on the appropriate course of action for each patient [95].

AI’s ability to streamline clinical workflows extends beyond image analysis and triage. By integrating with EHR systems, AI can automate various administrative tasks, such as data entry, documentation, and appointment scheduling [2,21]. This integration enhances the overall efficiency of clinical operations and reduces the administrative burden on healthcare providers. For instance, AI algorithms can extract relevant information from EHRs and populate patient records automatically, ensuring that the data are accurately recorded and readily available for clinical decision-making. Additionally, AI can optimize appointment scheduling by predicting no-show rates and adjusting schedules, accordingly, maximizing the utilization of clinical resources [21].

A notable example of AI optimizing workflow in ophthalmology is its application in glaucoma management. Glaucoma requires regular monitoring of intraocular pressure, visual fields, and optic nerve health [16]. AI-driven platforms can automate the analysis of visual field tests and OCT images, providing consistent and objective assessments [63]. These platforms can detect subtle changes in the optic nerve head and retinal nerve fiber layer, which are critical for early diagnosis and monitoring of glaucoma [17,64]. Moreover, AI systems can integrate data from multiple sources to provide a comprehensive risk assessment for each patient [22,73]. For example, combining IOP measurements, visual field data, and OCT results, AI can predict the risk of glaucoma progression and recommend personalized monitoring and treatment plans. This approach not only enhances the efficiency of clinical workflows but also improves patient outcomes by enabling early and targeted interventions.

## 6. Challenges and Ethical Considerations

### 6.1. Data Privacy and Security

The integration of AI in ophthalmology brings significant benefits but also raises critical concerns about data privacy and security. The confidentiality and integrity of patient data must be maintained to ensure trust and compliance with regulatory standards. This section explores the challenges associated with data privacy and security in AI-driven ophthalmology and the measures needed to address these issues. One of the primary concerns in AI-driven ophthalmology is ensuring that patient data remain confidential and secure [96,97]. AI systems often require access to large datasets, including retinal images, medical histories, and demographic information, to train algorithms and improve diagnostic accuracy [21]. The collection, storage, and processing of this sensitive information pose significant privacy risks. To ensure data confidentiality, robust encryption methods must be employed both in transit and at rest [97]. This means that data should be encrypted when they are being transmitted over networks and when they are stored in databases. Encryption ensures that unauthorized parties cannot access or tamper with the data, thereby maintaining its integrity [98]. Additionally, access controls must be strictly enforced to ensure that only authorized personnel can access sensitive patient data. This includes implementing multi-factor authentication (MFA) and role-based access control (RBAC) to limit access based on the user’s role and necessity. Regular audits and monitoring of access logs are also essential to detect and respond to unauthorized access attempts promptly [99].

Robust data governance frameworks are crucial for protecting sensitive information and ensuring compliance with regulatory standards, such as the General Data Protection Regulation (GDPR) in Europe, the Health Insurance Portability and Accountability Act (HIPAA) in the United States, and other national data protection laws [100,101]. These frameworks provide guidelines and policies for data collection, storage, processing, and sharing, ensuring that patient data are handled ethically and legally. Key components of an effective data governance framework include data minimization, data anonymization, data retention policies, consent management, and transparency and accountability [102,103]. Data minimization involves collecting only the data necessary for specific AI applications to reduce the risk of exposure [104]. Data anonymization involves removing or masking personally identifiable information (PII) to protect patient privacy while allowing for data analysis [105,106]. Data retention policies establish clear guidelines on how long data should be retained and ensure their secure disposal when no longer needed [107]. Consent management ensures that patients provide informed consent for the use of their data, with clear explanations of how it will be used and the benefits and risks involved [108].

Compliance with regulatory standards is essential for maintaining trust and ensuring that AI applications in ophthalmology adhere to legal and ethical guidelines. Regulations such as GDPR and HIPAA set stringent requirements for data protection, including the rights of individuals to access, correct, and delete their data, and the obligation of organizations to report data breaches promptly [100,101]. To comply with these standards, organizations must implement comprehensive data protection measures. These measures include conducting Data Protection Impact Assessments (DPIAs) to identify and mitigate potential privacy risks associated with AI applications; establishing breach notification procedures to detect, report, and respond to data breaches in a timely manner [109]; ensuring staff training and awareness in data protection principles and practices; and managing vendors to ensure that third-party vendors and partners comply with data protection standards and contractual obligations to safeguard patient data [110].

### 6.2. Bias and Fairness

AI algorithms, despite their transformative potential in ophthalmology, can inadvertently introduce biases that lead to disparities in patient care [111]. The primary sources of bias in AI systems include the training data, algorithm design, and operational implementation. Training data bias occurs when the datasets used are not representative of the broader population, such as a retinal image dataset dominated by images from a single demographic group, which may result in algorithms that perform inadequately on other demographic groups [111,112]. Algorithm design bias can stem from developer decisions regarding feature selection, model architecture, and hyperparameter tuning. Operational bias arises during the deployment and use of AI systems, where the interaction with clinical workflows and the interpretation of AI outputs by healthcare providers may introduce unintended biases [113].

To ensure fairness and generalizability in AI models, it is imperative to develop and validate these models using diverse and representative datasets. In ophthalmology, this means including retinal images from patients with diverse skin tones, ages, and underlying health conditions [114]. Bias detection and mitigation techniques should be employed during the training process to identify and address biases [112,114]. Fairness metrics can be used to assess the performance of AI models across different subgroups, and techniques such as reweighting or resampling data, adversarial debiasing methods, and incorporating fairness constraints in training can help reduce bias [112,115].

Robust validation of AI models on independent and diverse datasets not used during training is essential to ensure these models generalize well to new, unseen data. This involves using cross-validation techniques and conducting external validation studies to test the model’s performance across various population groups [116]. Continuous monitoring and updating of AI systems are necessary to maintain their fairness and reliability over time [117]. This ongoing process helps to identify new biases as they emerge and adjust the models accordingly.

### 6.3. Integration into Clinical Practice

Integrating AI into existing clinical workflows in ophthalmology poses several challenges, particularly concerning interoperability and user acceptance. For AI tools to be effectively utilized, they must seamlessly integrate with the current healthcare infrastructure, including EHRs and other clinical systems [22,71]. This requires robust interoperability standards to ensure that AI systems can communicate and exchange data with these existing platforms without disruption.

One of the primary hurdles in integrating AI into clinical practice is ensuring that healthcare professionals are adequately trained to use these tools. This involves not only technical training but also educating clinicians on the benefits and limitations of AI systems [71,74,118,119]. By providing comprehensive training programs, healthcare organizations can help clinicians become proficient in using AI tools, thereby improving user acceptance and confidence in these technologies [71,118]. Addressing concerns about job displacement is also critical. AI should be positioned as a tool that enhances the capabilities of healthcare professionals rather than replacing them [71]. By automating routine tasks and providing decision support, AI can free up clinicians to focus on more complex and patient-centric activities, ultimately improving patient care.

Successful implementation of AI in ophthalmology requires collaborative efforts between technologists and clinicians [84]. This collaboration is essential for designing AI solutions that are both user-friendly and clinically relevant. Clinicians can provide valuable insights into the practical challenges and needs of clinical practice, while technologists can offer expertise in developing sophisticated AI algorithms and systems [84,120]. Together, they can create AI tools that fit seamlessly into clinical workflows and address real-world clinical problems. For example, incorporating feedback from ophthalmologists during the development phase can lead to the creation of AI tools that are intuitive and tailored to the specific needs of eye care.

Furthermore, it is important to engage in continuous dialogue with all stakeholders, including patients, to ensure that AI systems meet their needs and expectations [22,121,122]. Patient education and transparency about how AI is used in their care can enhance trust and acceptance [123]. By fostering a culture of collaboration and open communication, healthcare organizations can facilitate the integration of AI into clinical practice, ensuring that these technologies are embraced and effectively utilized to enhance patient outcomes.

## 7. Future Directions

### 7.1. Enhanced AI Models

The future of AI in ophthalmology lies in the development of more sophisticated models capable of handling multimodal data, which include not only imaging data but also genetic information, patient histories, and other relevant clinical data. Such comprehensive models have the potential to revolutionize our understanding of disease mechanisms and enhance the precision of treatment responses. One promising direction for future research is the integration of genetic data with traditional imaging and clinical data [124]. By combining these diverse data types, AI models can offer a more holistic view of a patient’s health, potentially identifying genetic predispositions to certain eye diseases and predicting how these conditions might progress over time. For instance, incorporating genetic information could help identify patients at higher risk for conditions like AMD or DR long before clinical symptoms appear, allowing for earlier and more targeted interventions [42,52]. Additionally, leveraging patient history data, including previous treatments, outcomes, and other health conditions, can further refine AI models. This approach enables the creation of personalized treatment plans that account for a patient’s unique medical background, improving the accuracy and effectiveness of care. For example, in managing glaucoma, an AI system that considers a patient’s comprehensive medical history could better predict disease progression and suggest personalized treatment adjustments, enhancing overall patient outcomes [64].

Developing advanced AI models will require collaboration across multiple disciplines [125], including ophthalmology, genetics, bioinformatics, and computer science. Researchers will need to address several technical challenges, such as ensuring data interoperability, managing large and complex datasets, and developing algorithms that can seamlessly integrate and analyze multimodal data. Moreover, ethical considerations, such as patient consent for the use of genetic data and the potential for genetic discrimination, must be carefully managed to ensure patient trust and regulatory compliance. By focusing on these future directions, the field of ophthalmology can harness AI’s full potential, leading to more precise diagnoses, personalized treatments, and, ultimately, better patient outcomes. As AI models become increasingly sophisticated and capable of integrating diverse data sources, they will play a crucial role in advancing ophthalmic care and research.

### 7.2. Global Health Initiatives

AI-driven teleophthalmology services hold immense potential for addressing global eye health challenges, particularly in low-resource settings. These services leverage AI technologies to screen for and diagnose eye diseases remotely, thereby overcoming geographical and infrastructural barriers that often limit access to quality eye care. By expanding the reach of teleophthalmology, AI can play a pivotal role in improving eye health outcomes on a global scale. In low-resource settings, the scarcity of trained ophthalmologists and advanced medical facilities significantly hampers the delivery of eye care. AI-driven teleophthalmology can mitigate these issues by providing accurate, real-time screening and diagnostic capabilities via mobile devices and internet platforms. For instance, AI algorithms can analyze retinal images taken with portable fundus cameras and identify signs of DR, glaucoma, or other eye conditions with high accuracy. Patients in remote areas can then receive timely referrals and follow-up care, which is critical for preventing vision loss and managing chronic eye diseases.

To effectively deploy AI solutions in low-resource settings, collaborative efforts between governments, NGOs, and technology companies are essential. Governments can play a crucial role by creating supportive policies and frameworks that facilitate the integration of AI in healthcare systems [126]. This includes investing in digital infrastructure, ensuring data privacy and security, and providing funding for AI-based health initiatives. NGOs, which often work on the ground in underserved communities, can help implement and scale AI-driven teleophthalmology programs. Their deep understanding of local health challenges and trust within the communities they serve make them valuable partners in these initiatives. NGOs can assist in training local healthcare workers to use AI tools, raising awareness about the importance of eye health, and facilitating the logistics of teleophthalmology services. Technology companies bring the necessary expertise in AI development and deployment. By collaborating with healthcare providers and NGOs, they can tailor AI solutions to meet the specific needs of different populations. For example, companies can develop user-friendly AI applications that are accessible to healthcare workers with varying levels of technical expertise. Moreover, these companies can provide ongoing technical support and updates to ensure that AI systems remain effective and up to date with the latest medical standards and practices.

The expansion of AI-driven teleophthalmology services can significantly improve access to quality eye care worldwide. In regions where traditional healthcare infrastructure is lacking, these services offer a practical and scalable solution to bridge the gap. For example, in rural and underserved urban areas, AI-powered mobile clinics can provide essential eye care services, reducing the burden of travel for patients and making it easier for them to receive timely and effective treatment. Furthermore, by enabling early detection and intervention, AI-driven teleophthalmology can help reduce the prevalence of preventable blindness and vision impairment. Early diagnosis of conditions such as DR or glaucoma can lead to better management and treatment outcomes, ultimately preserving vision and enhancing the quality of life for patients. This proactive approach not only benefits individual patients but also alleviates the broader economic and social burden associated with vision loss.

## 8. Limitations of the Review

While this narrative review provides a comprehensive overview of current AI applications in ophthalmology, it has several limitations. First, due to language constraints, only articles published in English were included, which may have led to the exclusion of relevant studies from key regions such as Europe, China, and India where AI research is rapidly emerging. Although modern translation tools (e.g., Google Translate) are available, their use was not employed in this review, potentially limiting the scope of the evidence considered. Second, as a narrative review, the quality of the included articles was not formally evaluated using systematic criteria such as a PICO strategy, which is essential for assessing the strength of evidence in systematic reviews. Consequently, the conclusions drawn from this review may lack the rigorous quality appraisal that is necessary for evidence-based practice. Future work should consider incorporating non-English literature and employing systematic quality assessment methods to further enhance the robustness and generalizability of the findings.

## 9. Conclusions

AI is poised to revolutionize ophthalmology by enhancing diagnostic accuracy, personalizing treatment, and improving service delivery. The application of AI in ophthalmology encompasses a wide range of innovations, from advanced diagnostic tools that can detect conditions like DR, age-related macular degeneration, and glaucoma with remarkable precision to personalized treatment plans that optimize therapeutic outcomes and reduce costs. Additionally, AI-driven surgical tools and teleophthalmology services are making high-quality eye care more accessible, particularly in underserved and remote areas. Despite these promising advancements, several challenges need to be addressed to fully integrate AI into ophthalmic practice. Data privacy and security remain paramount concerns, necessitating robust encryption methods, stringent access controls, and comprehensive data governance frameworks. Addressing algorithmic biases is also crucial to ensure that AI systems provide equitable care across diverse patient populations. Furthermore, interoperability issues and the need for user acceptance highlight the importance of training healthcare professionals and designing user-friendly AI solutions.

The potential benefits of AI in ophthalmology are immense. By continuing to invest in research and development, fostering ethical practices, and encouraging collaborative efforts between technologists, clinicians, and policymakers, the ophthalmic community can overcome these challenges. Such collaborative efforts are essential to develop and deploy AI systems that are both effective and ethically sound. As AI technology evolves, it holds the promise of transforming ophthalmic care, leading to better patient outcomes, more efficient clinical workflows, and broader access to high-quality eye care services globally. By harnessing the power of AI, we can make significant strides toward improving vision health and preventing blindness on a global scale.

## Figures and Tables

**Table 1:** AI in ophthalmic diagnosis.

Condition	AI Technology	Clinical Application	Advantages	Challenges
DR	CNNs	Automated screening from retinal images	High diagnostic accuracy, reduced screening time	Data privacy, algorithmic bias, integration with EHR
AMD	DL	Classification of AMD stages from OCT images	Early detection, improved patient outcomes	Training in diverse datasets, regulatory compliance
Glaucoma	AI algorithms for OCT and visual field analysis	Early diagnosis and monitoring through automated analysis of OCT and visual fields	Timely intervention, reduced vision loss	Data accuracy, handling large datasets
Cataract	ML models	Diagnosis and grading of cataracts from slit-lamp images	Automated assessment, standardized grading	Integration into clinical workflows, user training
RVO	DL models	Detection and classification of RVO from OCT images	Accurate diagnosis, reduced need for manual analysis	Ensuring model generalizability, data sharing
Retinitis Pigmentosa	Genetic data integration with imaging AI	Combined genetic and imaging analysis for early diagnosis	Comprehensive insights into disease mechanisms	Ethical considerations, data privacy
Keratoconus	AI for corneal topography	Early detection from corneal topography images	Early intervention, improved treatment outcomes	Ensuring algorithm fairness, handling complex data
Ocular Surface Diseases	ML and image analysis	Detection and monitoring of ocular surface conditions from slit-lamp and tear film images	Improved patient management, reduced workload	Data integration, maintaining data privacy
Uveitis	AI for imaging and clinical data	Diagnosis and monitoring from multimodal imaging and clinical data	Early detection, tailored treatment plans	Data complexity, ensuring unbiased algorithms

Abbreviations—AI: artificial intelligence; DR: diabetic retinopathy; CNNs: convolutional neural networks; EHR: electronic health records; AMD: age-related macular degeneration; DL: deep learning; OCT: optical coherence tomography; ML: machine learning; and RVO: retinal vein occlusion.

**Table 2:** AI in ophthalmology service delivery.

Service Area	AI Technology	Key Systems/Programs	Clinical Application	Advantages	Challenges
Screening Programs	AI-powered screening tools	EyeArt, IDx-DR, mobile AI apps	Early detection of eye diseases, such as DR and glaucoma	Increased access, early detection, reduced workload	Data privacy, ensuring accuracy in diverse settings
Teleophthalmology	AI in telemedicine platforms	Retina-AI, telehealth initiatives	Remote diagnosis and monitoring of eye conditions	Access to care in remote areas, timely referrals	Technology access, maintaining data security
Workflow Optimization	AI for task automation	AI-driven triage and scheduling	Automating image analysis, patient triage, and scheduling	Improved efficiency, allowing for focus on complex cases	Interoperability with existing systems, user acceptance
Patient Monitoring	AI in wearable devices	Smart contact lenses, AI apps	Continuous monitoring of eye conditions	Real-time data, proactive management	Data management, patient adherence
Decision Support Systems	Clinical decision support AI	IBM Watson, Google Health AI	Assisting in diagnosis and treatment planning	Enhanced decision-making, personalized care	Trust in AI recommendations, integration into workflow
Resource Allocation	AI for resource management	Hospital management AI tools	Optimizing use of medical resources and staff scheduling	Cost savings, improved resource utilization	Implementation costs, training staff
Patient Engagement	AI chatbots and virtual assistants	Chatbots for appointment scheduling, symptom checking	Enhancing patient communication and education	Improved patient satisfaction, reduced administrative burden	Accuracy of AI responses, patient data privacy

Abbreviations—AI: artificial intelligence; DR: diabetic retinopathy.
